# Enhancing ovarian cancer immunotherapy through TAGLN2-modified CAR-T cells

**DOI:** 10.1016/j.gendis.2026.102082

**Published:** 2026-02-13

**Authors:** Fang Yang, Hanqi Lou, Chun Zhao, Shiwei Duan, Yongming Xia

**Affiliations:** a Department of Hematology, Yuyao People's Hospital of Zhejiang Province, The Affiliated Yangming Hospital of Ningbo University, Yuyao, Zhejiang 315400, China; b Department of Clinical Medicine, School of Medicine, Hangzhou City University, Hangzhou, Zhejiang 310015, China

In a 2024 issue of *Nature*, Juan R. Cubillos-Ruiz and his team reported groundbreaking findings demonstrating that TAGLN2 supports the functional maintenance of protective CD8^+^ T cells by coordinating lipid metabolism.^1^ Their work proposed a novel strategy to enhance the efficacy of adoptive T cell immunotherapy against invasive solid tumors.

Ovarian cancer is among the most common malignancies affecting women, with high-grade serous ovarian cancer (HGSOC) representing one of the most aggressive subtypes. Chemotherapy remains a primary treatment for HGSOC; however, recurrence and drug resistance remain intractable clinical challenges, making immunotherapy a promising complementary approach.

T cells are central to antitumor immunity, but within the ovarian cancer tumor microenvironment (TME), their function is often suppressed, weakening immunotherapeutic responses. Notably, CD8^+^ T cells in ascites, tumor tissues, and peripheral blood of patients with HGSOC exhibit impaired lipid uptake. Even when the key lipid transporter fatty-acid-binding protein 5 (FABP5) is overexpressed, this defect is not corrected, indicating a deeper regulatory dysfunction.^2^ Previous studies have also demonstrated that nutrient scarcity and oxidative stress in the ovarian cancer microenvironment initiate a cascade of events: they induce endoplasmic reticulum (ER) stress in T cells, leading to activation of theinositol-requiring enzyme-1α (IRE1α)- X-box binding protein 1 (XBP1) signaling pathway. This pathway represses T cell glycolysis and fatty acid oxidation, driving effector T cells into an exhausted state and further compromising antitumor immunity.^3^

Transgelin 2 (TAGLN2), an actin-binding protein from the calponin family, is critical for T cell lipid metabolism and sustains the TAGLN2–FABP5 axis to enhance solid tumor immunotherapy efficacy. Activation of the XBP1 pathway in the ovarian cancer TME initiates an endoplasmic reticulum stress response wherein IRE1α removes a 26-nucleotide segment from XBP1 mRNA—a key step in generating the functional, spliced transcription factor isoform spliced XBP1 (XBP1s). The resulting XBP1s protein binds to the non-coding sequence CNS1 located in the 5′ promoter region of the TAGLN2 gene, ultimately repressing its expression. This suppression prevents FABP5 from translocating to the T cell surface, trapping it in the cytoplasm and thereby impairing T cell function.

The researchers hypothesized that restoring TAGLN2 expression could reinvigorate CD8^+^ T cell antitumor activity. They engineered chimeric antigen receptor (CAR) T cells to overexpress a modified version of TAGLN2, rendering them resistant to tumor-induced endoplasmic reticulum stress. These TAGLN2-enhanced CAR-T cells retained function and exhibited superior control of metastatic disease progression and extended survival in mouse models of metastatic ovarian cancer. This finding validates TAGLN2 as a promising target for ovarian cancer immunotherapy, with significant preclinical therapeutic efficacy (Fig. 1).

**Figure 1:**
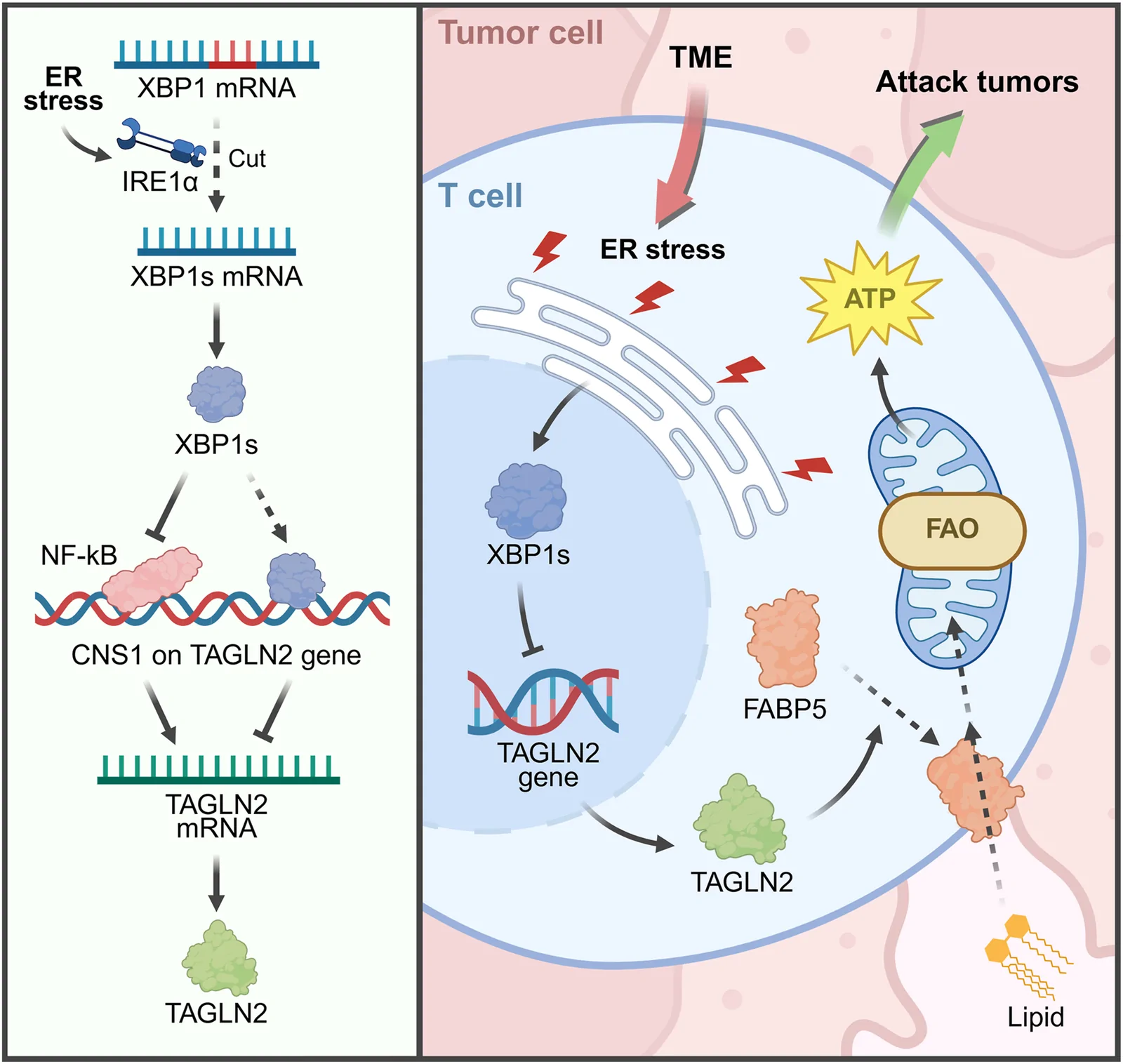
TAGLN2-mediated lipid metabolism supports T cell bioenergetics and effector function in the tumor microenvironment (TME). Within the TME, endoplasmic reticulum (ER) stress in T cells activates IRE1α, which in turn splices a specific nucleotide fragment from XBP1 mRNA, producing the active transcription factor XBP1s. XBP1s binds to the non-coding sequence CNS1 located in the 5′ promoter region of the TAGLN2 gene, directly repressing its transcription. Simultaneously, XBP1s also inhibits NF-κB-mediated transcriptional activation of TAGLN2. TAGLN2 promotes the translocation of FABP5 from the cytoplasm to the cell membrane, facilitating the uptake of extracellular lipids. These lipids undergo fatty acid oxidation (FAO), fueling mitochondrial respiration and generating ATP. This metabolic support equips T cells with the energy required to execute effective energy-intensive immune responses within the TME. Abbreviations: TME, tumor microenvironment; IRE1α, inositol-requiring enzyme-1α; XBP1, X-box binding protein 1; XBP1s, spliced XBP1; TAGLN2, transgelin 2; FABP5, fatty-acid-binding protein 5; FAO, fatty acid β-oxidation; ATP, adenosine triphosphate.

CAR-T cell therapy, a revolutionary adoptive cell transfer (ACT) approach, involves *ex vivo* collection, expansion, genetic engineering of patient immune cells and reinfusion for targeted cancer cell elimination. It has achieved remarkable success in hematologic malignancies, but its application to solid tumors is limited by multiple barriers: the immunosuppressive TME, poor CAR-T cell infiltration, severe adverse effects, limited *in vivo* persistence and the scarcity of tumor-specific antigens.

In ovarian cancer CAR-T therapy, several tumor-associated antigens have been explored, including mesothelin (MSLN), mucin 16 (MUC16), human epidermal growth factor receptor 2 (HER2), and folate receptor α (FRα). Research in this area is rapidly advancing. Cubillos-Ruiz's team further innovated by placing TAGLN2 cDNA downstream of the chimeric endocrine receptor (CER) construct, enabling CER-T cells to express high levels of TAGLN2. This modification enhanced T cell infiltration into ovarian tumors and improved their antitumor efficacy.

CER-T therapy, also referred to as endocrine receptor-targeted CAR-T therapy, represents a novel approach in solid tumor immunotherapy. This strategy leverages the follicle-stimulating hormone receptor (FSHR)—commonly expressed in ovarian tissue and tumor vasculature—as a target, enabling more precise tumor targeting and opening new avenues for CAR-T treatment of FSHR-expressing cancers.^4^

Despite promising preclinical results, TAGLN2-overexpressing CAR-T cells have limitations: their *in vivo* persistence in mice is only approximately one week, raising questions about long-term efficacy and potential adverse effects. Moreover, the physiological differences between mouse models and the human TME necessitate cautious interpretation. Ovarian cancer's complexity means monotherapy cannot fully overcome treatment resistance. The ovarian cancer TME is enriched with tumor-associated macrophages (TAMs), which recognize cytokine signals from activated CAR-T cells and secrete pro-inflammatory cytokines such as interleukin-6 (IL-6) and tumor necrosis factor-α (TNF-α), triggering a “cytokine storm” that exacerbates CAR-T-induced cytokine release syndrome (CRS), leading to fever, hypotension and multi-organ damage.

Therefore, when applying TAGLN2-modified CAR-T cells in ovarian cancer treatment, the TAM-mediated amplification of CRS is a core safety concern. Mechanistically, this application presents a potential contradiction: on one hand, the enhanced anti-tumor function of CAR-T cells by TAGLN2 may be accompanied by increased secretion of interferon-γ (IFN-γ), further activating TAMs and promoting pro-inflammatory cytokines release, forming a “CRS exacerbation loop”. On the other hand, TAGLN2 can reprogram T cell metabolism (e.g., by optimizing lipid uptake and fatty acid oxidation efficiency) and regulate the cytokine secretion profile of CAR-T cells—for instance, potentially reducing the excessive release of pro-inflammatory cytokines or moderately increasing the expression of anti-inflammatory factors, thereby alleviating CRS to some extent.

The core of this contradiction lies in the dual regulatory effects of TAGLN2 on CAR-T cells: it enhances anti-tumor activity through functional augmentation while modulating cytokine release via metabolic reprogramming, with the two exerting opposing influences on CRS. Thus, the overall regulatory trend of TAGLN2 modification on CRS requires further experimental validation in ovarian cancer-specific preclinical models (such as orthotopic xenograft mouse models), through systematic monitoring of dynamic cytokine changes, TAM activation status, and animal survival indicators.

As such, future research should focus on optimizing CER-T approaches, improving durability, and reducing side effects for more effective and sustainable therapeutic outcomes. Recent studies focusing on ovarian cancer treatment have confirmed that allogeneic CAR-NKT cells demonstrate significant core advantages over conventional CAR-T cells in treating ovarian cancer: they not only exhibit superior efficacy in inhibiting tumor progression and overcoming tumor immune resistance but also possess unique potential in reducing the risk of treatment-related adverse reactions. This finding provides robust experimental support for addressing key challenges faced by CAR-T cell therapy in ovarian cancer, such as tumor escape and insufficient *in vivo* persistence.^5^

Therefore, integrating the TAGLN2 modification strategy into the CAR-NKT cell system is expected to combine two major advantages—leveraging TAGLN2's metabolic regulatory functions (e.g., optimizing lipid uptake and fatty acid oxidation to sustain cell activity) while capitalizing on the inherent “efficacy-safety” dual characteristics of CAR-NKT cells. This integrated approach aims to achieve two key objectives: first, to extend the survival time of cells within the TME, thereby enhancing sustained anti-tumor capacity; and second, to concurrently reduce the risk of CRS and improve treatment safety. This combined strategy may emerge as a promising new research direction with high clinical translation potential for ovarian cancer immunotherapy.

In conclusion, upregulation of TAGLN2 in the ovarian cancer TME has shown significant promise in restoring CD8^+^ T cell function. TAGLN2-modified CAR-T cell therapy represents an exciting frontier in ovarian cancer treatment, offering a solid scientific foundation for the development of novel immunotherapeutic strategies. With continued research and innovation, TAGLN2 and CAR-T technologies may also find applications in other solid tumors, potentially transforming the therapeutic landscape and offering new hope to patients with otherwise intractable cancers.

## CRediT authorship contribution statement

**Fang Yang:** Writing – original draft. **Hanqi Lou:** Visualization. **Chun Zhao:** Conceptualization. **Shiwei Duan:** Writing – review & editing. **Yongming Xia:** Writing – review & editing.

## Funding

This study was supported by Zhejiang Province Basic Public Welfare Research Project, China (No. LBY24H080001).

## Conflict of interests

The authors declare that they have no competing interests.
